# Effect of Different Compatibilization Systems on the Rheological, Mechanical and Morphological Properties of Polypropylene/Polystyrene Blends

**DOI:** 10.3390/polym12102335

**Published:** 2020-10-13

**Authors:** Martina Seier, Sascha Stanic, Thomas Koch, Vasiliki-Maria Archodoulaki

**Affiliations:** Institute of Materials Science and Technology, TU Wien, Getreidemarkt 9, 1060 Vienna, Austria; sascha.stanic@tuwien.ac.at (S.S.); thomas.koch@tuwien.ac.at (T.K.); vasiliki-maria.archodoulaki@tuwien.ac.at (V.-M.A.)

**Keywords:** polypropylene, polystyrene, compatibilization, blends

## Abstract

The influence of reactive processing, non reactive and reactive copolymers on immiscible polypropylene (PP)–polystyrene (PS) blends with varying PS concentrations (10 wt.% and 25 wt.%) was evaluated by mechanical (tensile and tensile impact), rheological (melt flow rate, extensional and dynamic rheology) and morphological (scanning electron microscopy) analysis. As an extended framework of the study, the creation of a link to industrial applicable processing conditions as well as an economically efficient use of compatibilzing agent were considered. For radical processed blends, a high improvement in melt strength was observed while non reactive copolymers exhibited a pronounced increase in toughness and ductility correlated with overall best phase homogeneity. Conversely, the influence of the reactive copolymer was quite different for the varied PS concentrations not allowing the assumption of a specific trend for resulting blend properties, but nevertheless in the case of a lower PS concentration the tensile impact strength exceeded the value of virgin PP. Since PS and PP are widely used, the findings of this work could not only be relevant for the generation of more versatile blends compared to virgin components but also for recycling purposes, allowing the enhancement of specific properties facilitating the production of more valuable secondary materials.

## 1. Introduction

Melt blending of two or more polymers provides a rapid method for endowing materials with a broader range of properties while overcoming the disadvantages of selected individual components [1]. Due to limited compatibility leading to inhomogeneous structures directly influencing the physical properties and therefore the overall performance of the material, the successful generation of combined-property polymers is not easily achieved without drawbacks [2]. Phase separation behavior is greatly influenced by interfacial tension between the polymers, their density and viscosity ratios [3,4]. To overcome that problem, different compatibilizing systems have been extensively studied over the last years; either motivated by the idea of generating entirely new materials [5,6,7] or the improvement of recyclability of mixed polymer waste [8,9].

Three commonly known methods to achieve compatibilization of polymer blends with poor miscibility are [10]:

- Copolymers;
- reactive graft copolymers;
- radical processing.

Various copolymers can be added to incompatible polymer mixtures and, being able to react with both chain ends for higher bonding strength, act as a bridge structure. Especially thermoplastic elastomers, such as ethylene-propylene-diene (EPDM) or styrene-ethylene-butene-styrene block copolymer (SEBS), have proven to be very effective for this purpose [11,12,13,14].

Many commercial polymers like polyolefins show relatively low reactivity. Radical processing is used to enhance the reactivity of different materials involving the formation of free radicals which can lead to scission and recombination of polymer chains. This makes the coupling of immiscible phases via single junctions possible [10]. However, controlling the occurring reactions is crucial as the presence of radicals might not only lead to branching but also cross-linking reactions or accelerated oxidative degradation and random chain scission [15,16].

Compatibilization with reactive graft copolymers, like maleic anhydride containing ones, combines elements from both procedures mentioned above. These copolymers carry additional functional groups like anhydride, carboxylic acid or epoxy enhancing reactivity during melt blending [17]. A combination of non reactive and reactive coupling actions of the incompatible polymer phases might subsequently be initiated leading to complex block or graft structures located at the interface [18]. Improvements of morphology and mechanical properties have been reported by many authors for various materials [19,20,21].

For the concrete case of polypropylene (PP)–polystyrene (PS) blends, Rhagu et al. [22] investigated the influence of three different non reactive thermoplastic elastomers. They found that the size of particles of the dispersed phase was significantly reduced in all cases indicating improved compatibility whereas mechanical and rheological properties were strongly dependent on type and amount of used compatibilizer. Mustafa et al. [23] used an aromatic vinyl grafted PP and observed improvements in morphology in addition to mechanical behavior. In was also shown that MFR. values of PP rich blends exhibited a negative deviation compared to virgin PP, which was related to the interparticle interaction and morphological deformability. Reactive extrusion of PP–PS blends in the presence of peroxide and different stabilizers was performed by Xie [24] et al. and Li et al. [25]. In both cases, PP-g-PS graft polymers were successfully formed and a decrease of the average dispersed particle size was observed via microscopy.

However, the successful production of blends with balanced properties is critical, since on the one hand the concentration of the compatibilizer plays an important role and on the other hand the thermal-oxidative material degradation is favored with increasing process temperature [26,27]. Nevertheless, specific processing temperatures are required to meet industrial standards. Regarding the amount of added compatibilizer, Brostow et al. reported that the morphology of PP/PS blends, especially the size of the dispersed droplets, is influenced by the addition of styrene-ethylene-butylene-styrene block copolymer up to 5%. At higher concentrations, no more changes in the morphology could be observed which might be due to a certain saturation of the polymer system [28,29]. Despite the many studies that have been performed in this field and especially improvements in elongation at break and increase of impact toughness being reported by various authors for different methods [30,31,32,33], no direct comparison of reactive extrusion, non reactive and reactive copolymers has yet been provided for blends containing PP and PS.

An evaluation of the effectiveness of various compatibilization methods and the consequent generation of PP–PS blends with balanced properties under industrial and economical suitable conditions could also be a chance to generate attractive recycling material of a rather unconventional polymer combination in the scope of a mechanical recycling process. This is especially relevant in the packaging sector where both materials are present (major PP and minor PS fraction) and the products have rather short shelf lives [34].

**Figure 1:**
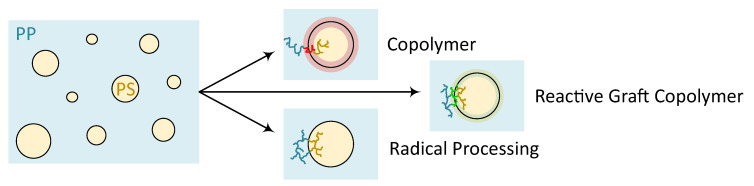
Schematic representation of possible compatibilization mechanisms of the different systems regarding the interface of matrix and dispersed phase.

With this kept in mind, the impact of three different compatibilization mechanisms schematically presented in Figure 1 on PP–PS blends with two polystyrene concentrations was investigated in this study. The concentration of compatibilizer was held low as on the one hand saturation is often reported in literature to occur over 5% and on the other hand due to economic attractiveness. To evaluate the overall performance of the generated blends, rheological, morphological and mechanical tests were performed.

## 2. Materials and Methods

### 2.1. Materials

Isotactic PP homopolymer (HC 600 TF) supplied by Borealis (Vienna, Austria) with a melt flow rate (MFR) of 2.8 g/10 min (230 ${}^{\circ }$C/2.16 kg) and atactic general purpose PS (PS GP 152) supplied by Synthos (Vienna, Austria) with an MFR of 2.5–3.5 g/10 min (200 ${}^{\circ }$C/5 kg) were used as the major components of the blends. PP and PS are both commercial grade polymers used in various thermoforming applications.

**Figure 2:**
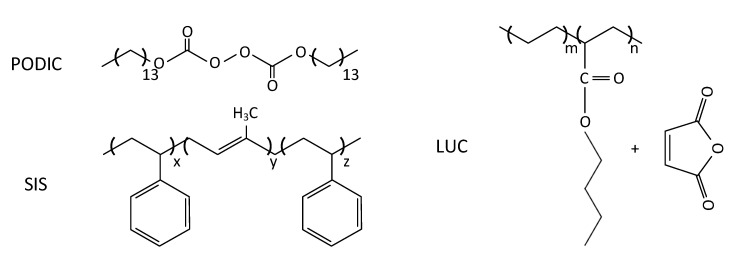
Chemical structures of the added supplements.

Polystyrene-block-polyisoprene-block-polystyrene (SIS) with an MFR of 3.0 g/10 min (200 ${}^{\circ }$C/5 kg) and a bound styrene content of 22 wt.% was purchased from Sigma-Aldrich (St. louis, MO, USA). A chemical modified ethylene butyl acrylate (EBA) copolymer grafted with maleic anhydride Lucofin^®^ 1492M HG (LUC) and an MFR of 2–5 g/10 min (190 ${}^{\circ }$C/2.16 kg) was supplied by Lucobit (Wesseling, Germany). These materials were used as non reactive and reactive copolymer compatibilizing agents. In addition, PODIC (Peroxan C126), a dimyristyl peroxydicarbonate (10 h half-life time at 48 ${}^{\circ }$C) supplied by Pergan (Bocholt, Germany) was used for reactive processing. Chemical structures of the supplements are shown in Figure 2.

### 2.2. Sample Preparation

In order to ensure continuous material feed during extrusion, SIS had to be shredded into smaller particles. This was achieved using a CryoMill (Retsch, Germany) at −196 ${}^{\circ }$C (liquid nitrogen cooled). Further, the specific blend components were pre mixed by hand and added directly to the extruder. Melt blending was conducted in an Extron single screw extruder (EX-18-26-1.5, Extron Engineering Oy, Akaa, Finland). The extruder has three individual heating zones, a screw diameter of 18 mm and a length/diameter ratio of 25:1. Melt blending was carried out at a temperature of 240 ${}^{\circ }$C (165/240/240 ${}^{\circ }$C from hopper to die) and a screw speed of 70 rpm. The blends prepared using this method are summarized in Table 1.

For further processing, the polymer strands generated from extrusion were shredded with a universal cutting mill (Pulverisette19, Fritsch, Germany) equipped with a 4 mm sieve insert.

### 2.3. Rheology

Characterization of the polymer melt was realized via dynamic and extensional rheology measurements as well as MFR determination.

Rheological specimens were processed by compression molding (Collin P 200 P, Ebersberg, Germany) at a temperature of 240 ${}^{\circ }$C and a pressure of 30 bar. Aluminum frames sandwiched between steel plates and separated by Teflon sheets were used to generate discs 25 mm in diameter and 1.2 mm in thickness and squares of dimension 60 mm × 60 mm × 0.8 mm, which were afterwards cut into 20 mm × 8 mm stripes for extensional rheology measurements.

For dynamic rheology, more specifically, frequency sweep tests, an MCR 301 rheometer (Anton Paar, Austria) equipped with a plate-plate system (1 mm gap size) and a CTD 450 heating chamber purged with nitrogen was used. During the experiment, temperatures were constantly held at 230 ${}^{\circ }$C and deformation raised logarithmically from 1% to 2% across a frequency range of 628 and 0.01 rad/s. The same instrument was also used for extensional rheology equipped with a SER-HPV 1 Sentmanat Extensional Rheometer (Xpansion instruments, Tallmadge, OH, USA). Samples were strained at three different rates (5 s${}^{-1}$, 1 s${}^{-1}$ and 0.1 s${}^{-1}$) at a temperature of 180 ${}^{\circ }$C. Corresponding start-up curves were measured with a plate-plate system using shear rates of 0.001 s${}^{-1}$ and 0.1 s${}^{-1}$ as shear rates.

MFR measurements were performed in accordance with DIN EN ISO 1133 method A under a load of 2.16 kg at 230 ${}^{\circ }$C using a manual testing device (MeltFloW basic, Karg Industrietechnik, Krailling, Germany).

### 2.4. Mechanical Properties

Mechanical properties were characterized via tensile and tensile impact strength testing. For that purpose, specimens were produced using a Haake Mini Lab II twin screw extruder in combination with a Haake Mini Jet II injection molding unit (Thermo Fisher Scientific, Waltham, MA, USA). Extrusion was carried out at a temperature of 240 ${}^{\circ }$C and a screw speed of 100 rpm. The mold temperature was set to 55 ${}^{\circ }$C and a pressure of 350 bar (10 s injection time) was used for the injection molding process.

Further, the specimens used for tensile impact strength testing (60 mm × 10 mm × 1 mm) were notched with a Notch-Vis (Ceast, Darmstadt, Germany) on both sides and tested with an Instron 9050 (2 J hammer and 15 g crosshead mass; Ceast, Darmstadt, Germany) in accordance with ISO 8256/1A.

Tensile testing of the injection molded specimen was performed in accordance with ISO 527-2-A5 at a speed of 10 mm/min using a Z050 testing frame (Zwick Roell, Ulm, Germany) equipped with a 1 kN load cell and an extensometer.

### 2.5. Morphological Characterization

For morphology analysis, the fractured surfaces of the tensile impact test specimens were analyzed using scanning electron microscopy (SEM; FEI Philips XL30, Hillsboro, OR, USA). Before measurement, the samples were coated with gold prior to imaging (Agar Sputter Coater B7340, Stansted, UK). SEM micrographs were further analyzed with a visualization software to generate standard distributions of the mean particle diameters. For that purpose, the area of 200 PS particles in each image was measured and the average diameters were calculated.

## 3. Results and Discussion

### 3.1. MFR

Due to the fact that the MFR values provided in data sheets are measured under different conditions, the melt flow rates of the individual components were additionally measured using the same parameters as the blends and the results are presented in Table 2.

**Figure 3:**
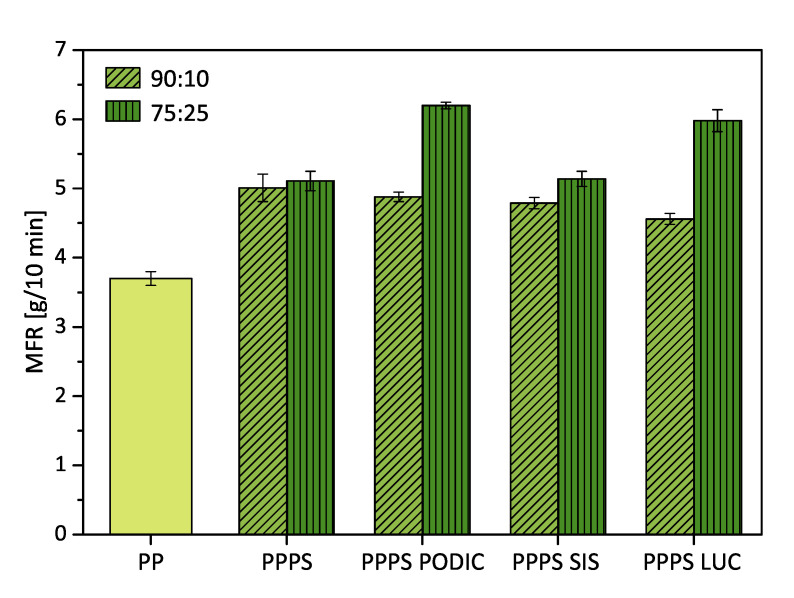
MFR values of virgin polypropylene (PP) and PP–polystyrene (PS) blends processed at 240 ${}^{\circ }$C; measured at 230 ${}^{\circ }$C.

Measurement of MFR values provide a good method to quantify the processability of polymer blends. Some authors have reported a slight decrease in MFR with increasing compatibilizer content due to better phase adhesion [35,36,37]. Similar behavior could be observed for the 90:10 blends with lower PS concentration in this study (Figure 3). Compared to virgin processed PP, the MFR is increased with the addition of PS but decreases with the addition of compatibilization agents.

Further, increase in the PS concentration resulted in no significant change in the MFR value of uncompatibilized blends. However, addition of compatibilizing agents lead to an increase of MFR.

At this point, it must be mentioned that the possible side reactions caused by reactive components can also clearly influence the flow properties. Highly long chain branched structures might result in an decrease of MFR while excessive chain scission promotes an opposite trend, which is a result of different chain mobility behavior [38,39]. As higher MFR values can be observed for both reactive components, at a higher PS concentration it seems possible that PS promotes chain scission reactions at the given temperature; furthermore, it correlates with compatibilizing efficiency.

SIS as the non reactive compatibilizer on the other hand shows quite stable behavior also at a higher PS concentration, which could be an indicator for overall best compatibility effect.

### 3.2. Dynamic Rheology

The dynamic deformation behavior of polymer melts is known to be a powerful tool to predict processability of materials in different manufacturing processes. In addition, the rheological properties are also very sensitive to changes in blend morphology and interaction between the polymer matrix and the dispersed phase [4].

**Figure 4:**
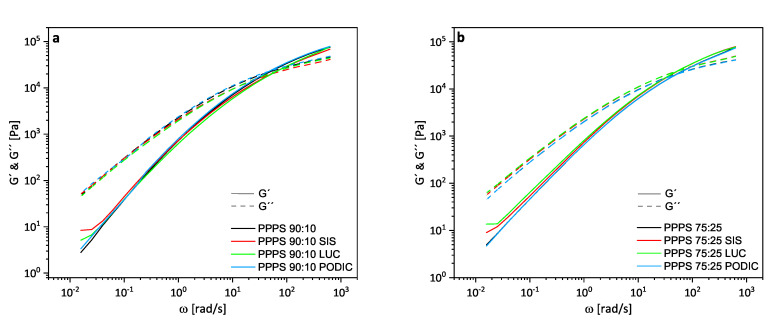
Storage and loss modulus (dashed) curves of PP–PS 90:10 (**a**) and PP–PS 75:25 (**b**) blends; measured at 230 ${}^{\circ }$C.

Figure 4 shows the storage modulus G′ and loss modulus G″ curves with respect to the angular frequency of the PP–PS blends. Both of these values provide important information about the response to stress of a polymer melt. Storage modulus relates to stiffness as it represents the amount of stored energy (elastic response), while loss modulus describes the amount of dissipated energy (viscous response) [40]. The G′ curves of the blends containing SIS and LUC tend to the formation of plateaus at low frequencies for both PS concentrations. This indicates the formation of strong networks in the polymer matrix leading to enhanced resistance against external forces at low frequencies due to a deviating relaxation process among dispersed particles. This is correlated with the presence of block copolymers and enhanced interfacial interaction [15,33,41,42,43]. Further, it can be seen that the elastic stress responses of SIS are more pronounced in the low frequency range for lower PS concentration compared to LUC while this behavior is kind of switched concerning PP–PS 75:25 blends. This might indicate that LUC as a reactive compatibilizer is able to provide better linkage of the increasing quantity of incompatible PS phase to the PP matrix.

Additionally, the crossover point of G${}^{\prime }$ and G${}^{\prime \prime }$ occurs at frequencies below 100 rad/s and can provide information about blend homogeneity and therefore compatibility. It displays the transition from domination of elastic to viscous behavior of the polymer melt associated with the amount of energy required to exceed the value provided by molecular or inter particle forces cohesion indicating material yielding [44]. Shift in G${}^{\prime }$ = G${}^{\prime \prime }$ is associated with changes in molecular weight (MW) and molar mass distribution (MMD). A crossover at lower frequencies indicates the presence of longer or branched molecules (longer relaxation times). Conversely, a vertical shift to lower G values is related to a broadening of MMD [45]. Values of crossover modulus $G_{c}$ and crossover frequency values $\omega_{c}$ are presented in Table 3. At lower PS concentrations, the addition of PODIC and SIS lead to an increase in molar mass while the opposite trend can be observed for PP–PS 75:25 blends. This supports the assumption from MFR measurements that higher PS concentration might promote chain scission during extrusion leading to an overall worse compatibility of blends. The blends containing LUC show different behavior as lower $\omega_{c}$ values were determined for the higher PS concentration and vice versa. The tendency for MMD change deducted from shifting of $G_{c}$ values are uniform across the various PS concentrations. All compatibilizing agents lead to broader distributions for 90:10 blends and MMD is narrowed for 75:25 blends.

**Figure 5:**
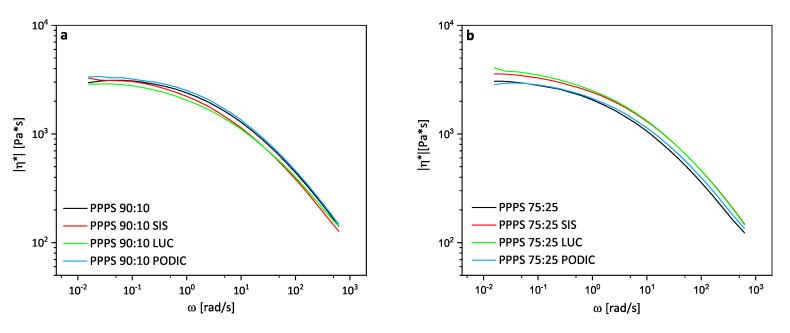
Complex viscosity as function of angular frequency of PP–PS 90:10 (**a**) and PP–PS 75:25 (**b**) blends; measured at 230 ${}^{\circ }$C.

Figure 5 displays the complex viscosity curves versus angular frequency of all blend compositions. SIS exceeds the viscosity value of uncompatibilized blends at low frequencies for both PS concentrations. Macaubas et al. [46] also reported an increase in complex viscosity for two different triblock copolymers used in PP–PS 90:10 blends indicating a compatibilizing effect. For different blend compositions, the same was also noticed by many other researchers in connection with non reactive copolymers [43,47,48,49].

In the case of maleic anhydride grafted copolymers used for compatibilization, various behaviors have been reported. Gwang et al. [50] used SEBS-g-MA and PP-g-MA as compatibilizer for PP/nylon (75:25) blends and observed higher complex viscosity values for SEBS-g-MA, but lower values for PP-g-MA compared to the virgin blend also indicating better and worse overall compatibility in terms of mechanical stability and phase morphology. Dobrovszky et al. [51] also used SEBS-g-MA but for the compatibilization of HDPE/PET blends at various compositions ratios. From ratios of 100/0 up to 75/25, the complex viscosity was lower compared to the uncompatibilized blend and with further increasing PET content the effect was inverted. In this case, the blend morphology was influenced differently showing finer dispersed particles at higher HDPE contents, while at high PET concentration the particle size was hardly influenced by the addition of SEBS-g-MA.

Regarding LUC and PODIC, the possible influence from chain scisson or branching resulting from reactivity actions during processing which might induce changes in viscosity must be considered. For 90:10 blends, the curve of PODIC is located above all other curves while for higher PS concentration it is located below LUC and SIS and in the low frequency range even below the uncompatibilized blend. In recently published work from our research group, similar behavior was observed for the reactive extrusion of PP at 240 ${}^{\circ }$C indicating the generation of a highly branched structure which was also confirmed by extensional rheology measurements [52]. A decrease in complex viscosity, as observed for 75:25 blends, is usually related to dominant chain scission during reactive extrusion with peroxides [25].

The damping factor or also tan(δ) defined as the quotient of the loss and storage modulus (G${}^{\prime \prime }$/G${}^{\prime }$) can give information about deviating relaxation time which is directly correlated to the macromolecular structure including changes in branch length, molecular weight or morphology [53].

**Figure 6:**
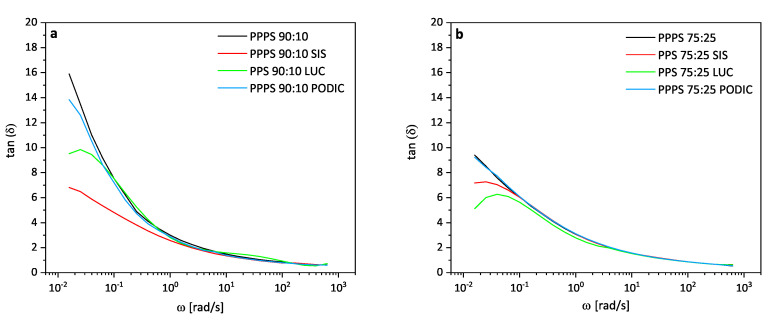
Loss factor of PP–PS 90:10 (**a**) and PP–PS 75:25 (**b**) blends; measured at 230 ${}^{\circ }$C.

A reduction in tan(δ) values at low frequencies can be related to a retardation in relaxation time indicating the enhancement of elastic deformation. Such behavior including the formation of a damping peak is displayed in Figure 6 (tan(δ) values versus frequency for all blends) for the blends containing LUC and SIS indicating more pronounced changes in morphology for these blends [43]. The uncompatibilized blends and PODIC almost show a completely viscous behavior for the low frequency range as values close to 90${}^{\circ }$ are reached for δ, whereas the effect is even more pronounced in the case of lower PS concentration.

Cheung et al. [54] also reported a direct correlation between melt elasticity and melt strength stating that the higher the melt elasticity the higher the melt strength. High elasticity is an important characteristic for materials used in processes in which the melt is excessively extended like fiber spinning, film blowing or foaming.

### 3.3. Extensional Rheology

In addition to tan(δ) plots indicating higher or lower elasticity, the performance of extensional rheology tests is a suitable method to get information about improved resistance of a polymer melt towards elongation before rupture which is referred to as strain hardening behavior [55].

**Figure 7:**
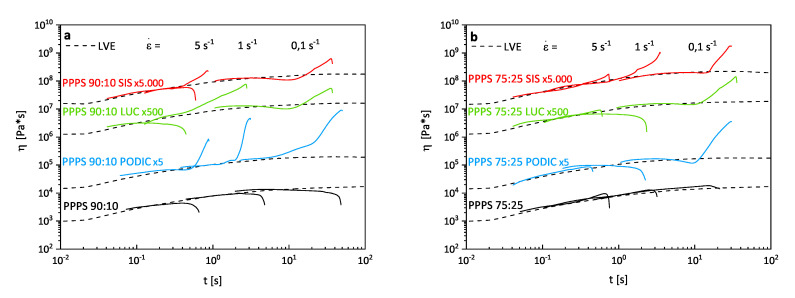
Extensional rheology curves of PP–PS 90:10 (**a**) and PP–PS 75:25 (**b**) blends; measured at 180 ${}^{\circ }$C.

Figure 7 details the results obtained from extensional rheology testing. The dashed lines represent the linear viscoelastic start-up curves (LVE) which are exceeded (strain hardening effect) by the extensional viscosity value-curves measured at different strain rates ($\dot{\varepsilon }$ = 5 s${}^{-1}$; 1 s${}^{-1}$; 0.1 s${}^{-1}$) or not. Since the start-up curves are measured as shear viscosity it needs to be considered that extensional viscosity and shear viscosity are not equivalent (extensional viscosity always exhibits higher values) but according to Trouton a value of 3 can be applied for correlation purposes [56].

In the case of virgin blends, no strain hardening effect can be observed for both PS concentrations which is common for linear polymers. According to Wagner et al. [57] the response to uniaxial extension is strongly related to chain length and long chain branched molecules indicating that the reactive extrusion with PODIC led to the formation of a significant quantity of long chain branches in the case of PP–PS 90:10 blends. For higher PS concentrations the strain hardening effect is lowered but still pronounced at a small strain rate. This may result from more pronounced scission reactions rather than recombination of polymer chains also indicated by the higher MFR values observed for this composition.

Stary et al. [3] observed an increase in elongational viscosity due to the stabilization of droplets against break up during flow for the compatibilization of PS–PE–LLD blends with 1 wt.% of styrene-butadiene-styrene triblock copolymer. This is further explained as an effect of stress not being fully transported from matrix to dispersed phase particles which might cause the quite equal strain hardening behavior of SIS for both PS concentrations. In recent studies, López-Barrón et al. [58] suggested that significant strain hardening in symmetrical and nonsymmetrical PP/PE blends caused by the addition of up to 5 wt.% poly(ethylene-cb-propylene) comb block copolymers. They concluded that the comb block molecules lead to interfacial stitching generating an elastic membrane also being responsible for the appearance of a G′ plateau at lower frequencies.

In the case of LUC as a reactive compatibilizer, the increase in extensional viscosity could either be due to the formation of branches, the influence of triblock copolymer or a combination of both.

For a more quantitative description of strain hardening effect and therefore melt strength, the strain hardening coefficient was calculated regarding to Equation (1) in which η(t) represents the maximum extensional viscosity at the corresponding strain rate and $\eta_{0}\left(t\right)$ describes the extensional viscosity of the LVE curve.

(1) $$SH=\frac{\eta (t)}{\eta_{0}\left(t\right)}$$

**Figure 8:**
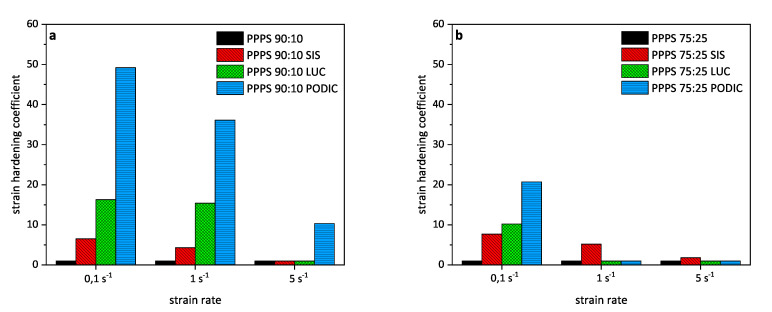
Strain hardening coefficients of PP–PS 90:10 (**a**) and PP–PS 75:25 (**b**) blends obtained from extensional rheology measurements.

Calculated values for all blends are provided in Figure 8. In the case of 90:10 blends for which strain hardening is generally more pronounced according to extensional rheology curves, PODIC obviously exhibits much higher values compared to the other compositions. For PODIC and LUC a decrease in strain hardening coefficient can be observed with increasing PS concentration while the blends containing SIS are hardly affected by a variation in PS content.

### 3.4. SEM

The morphology of binary blends in which the volume fraction of one polymer is dominant often occurs as droplets of the minor phase dispersed in the matrix of the major component [59]. Sundararaj et al. [60] describe the evolution of phase morphology in a co-rotation twin screw extruder as at the beginning of softening sheets of the dispersed phase are formed which grow as result of interfacial tension forces. At a certain point, the sheets merge into each other forming thin unstable ligaments which then break up due to sheer forces and form the dispersed phase droplets.

Detailed formation on the combined structure as well as the exact size and shape of particles are related to a complex mixture of impact factors like viscosity ratio, elasticity, polarity, interfacial adhesion, mixing and processing conditions [61,62]. Nevertheless, successful compatibilization has been reported by various authors to result in decrease of particle size and an overall more homogeneous structure for many polymer mixtures in various component ratios [63,64,65,66,67,68].

**Figure 9:**
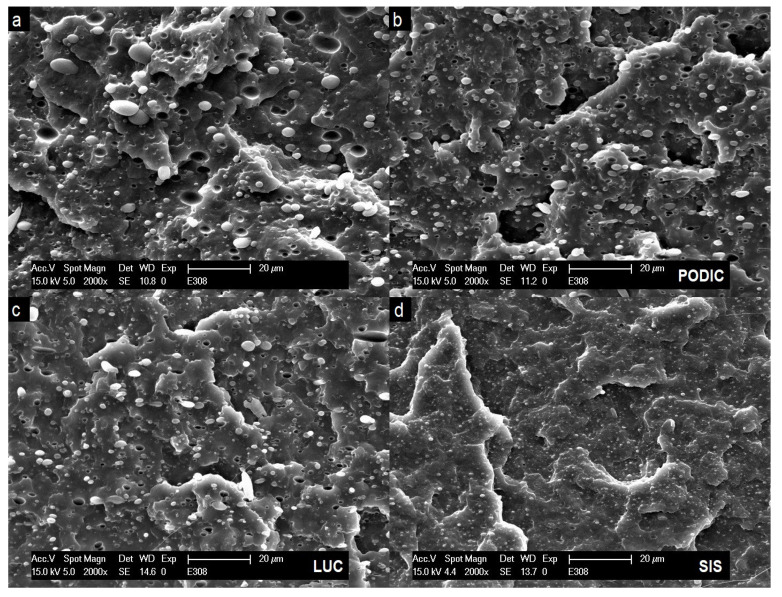
SEM images of PP–PS 90:10 blends: uncompatibilized (**a**), PODIC (**b**), LUC (**c**), SIS (**d**).

**Figure 10:**
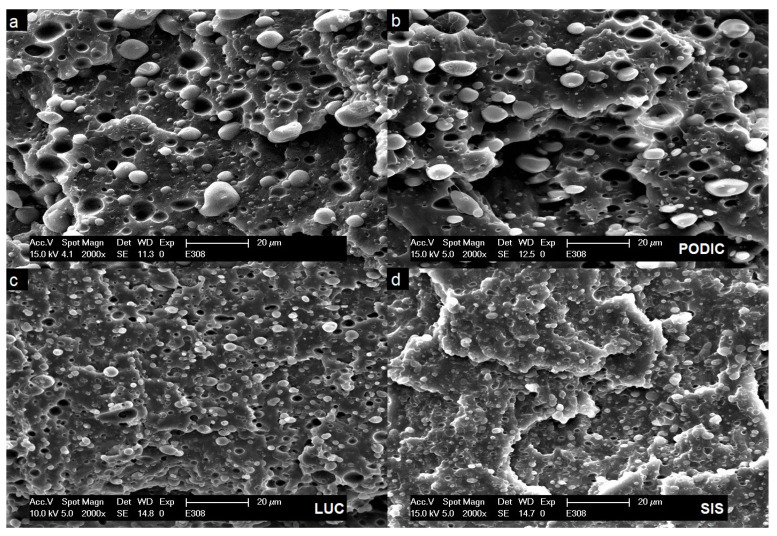
SEM micrographs of PP–PS 75:25 blends: uncompatibilized (**a**), PODIC (**b**), LUC (**c**), SIS (**d**).

Figure 9 and Figure 10 show the morphology of non compatibilized and compatibilized PP–PS blends at a 2000x magnification. A clear two-phase system is displayed for virgin blends with PS particles dispersed in the PP matrix related to high interfacial energy between the components [28]. Regarding to Escudie et al. [69], the interfacial tension values of polypropylene and polystyrene vary between 5–8–3.7 mN/m for a temperature region of 220–250 ${}^{\circ }$C, while Chapleau et al. [70] reported an interfacial tension of 17.4–14.6 mN/m between polypropylene and polycarbonate in the range of 225–250 ${}^{\circ }$C. In these cases, the difference in interfacial tension is related to lower and higher differences in polarity also being a relevant factor concerning the overall blend compatibility. Patterson et al. [71] did pioneer work regarding the compatibilization of polymers by developing that introducing a styrene hydrogenated butadiene(ethylenebutane) triblock copolymer led to a reduction in interfacial tension of approximately 4 mN/m from 5 to 1.1 mN/m in PP–PS blends.

It has further been reported by Datta et al. [72] that polymer blends need a stable dispersion with small droplets with diameters below a range of 0.3–3 μm. This is clearly not the case for the uncompatibilized blends as some particles exceed the size of 5 μm even in the case of lower PS concentration. Nevertheless, in the case of 90:10 blends, all applied compatibilizing systems lead to a successful decrease and more homogeneous distribution of PS particles while SIS clearly shows the finest dispersion. At higher PS concentrations the addition of SIS and LUC led to a similar decrease in particle size but for PODIC hardly any changes in morphology are visible. This is especially in good accordance with strain hardening behavior as the effect is drastically reduced for PODIC at higher PS concentrations.

As the surfaces of fractured tensile impact test specimen are displayed, the deformation of morphology under dynamic uniaxial stress can also be analyzed which might allow an assumption on possible toughening behavior. Under load, the PS particles are pulled out of the PP matrix leaving corresponding holes. This can be observed in the case of all blend compositions.

Furthermore, compared to uncompatibilized blends, no significant differences in particle shape or a deformation of the matrix between the particles is visible as compatibilizing agents are added meaning that no commonly occurring toughening effects like void formation or fibrillation can be seen [73].

However, a critical interparticle distance for toughening as well as a sharp brittle-tough transition which indicates that monodisperse and asymmetric particles result in a higher toughening effect than polydisperse and spherical ones was reported by Wu and Margolina [74].

**Figure 11:**
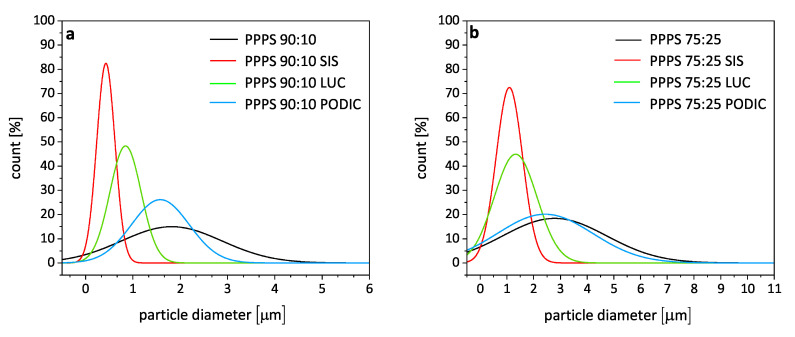
Normal distributions for calculated particle diameters of PP–PS 90:10 (**a**) and PP–PS 75:25 (**b**) blends.

For a better visualization of particle size distribution and average diameters for the different blends, normal distributions of particle size are given in Figure 11. Mean particle diameters ($d_{p}$) of 200 particles from every image were calculated according to Equation (2) assuming a corresponding spherical shape of droplets. $A_{p}$ refers to the real particle area measured with a visualization software tool.

(2) $$d_{p}={\left(\frac{A_{p}\cdot 4}{\pi }\right)}^{0.5}$$

For 90:10 blends, it is visible that the average mean particle diameter using PODIC is overall not much decreased but a narrower distribution is realized as especially really large particles >5 μm are reduced in size. As already assumed from SEM figures, the PP–PS 75:25 PODIC blend shows an almost identical average mean diameter value and standard distribution compared to the virgin blend. According to Wang et al. [66], an increase in branch length could improve the compatibilizing effect leading to finer dispersed morphologies which would also be in good agreement with extensional viscosity measurement results indicating more pronounced strain hardening and the existence of long chain branches.

SIS shows a considerable reduction of dispersed particles from an average mean diameter of 1.9 μm (uncompatibilized blend) to 0.4 μm being close to 80%. For 75:25 blends. the average value is reduced from 3.2 μm to 0.9 μm which corresponds to 72%. That is again in good agreement with the results from MFR and rheological analysis indicating that SIS exhibits fairly constant behavior across both PS concentrations. LUC shows the almost perfect contrary behavior as for 90:10 blends a reduction in particle size by 58% is observed and by 63% for 75:25 blends, respectively.

Regarding the general particle size of dispersed PS droplets in PP blends, various different values are given in literature being highly dependent on viscosity ratio [75], processing, as well as cooling temperature [76] and also mixing procedure [77]. Wang et al. [78] found diameters of 0.4–1.5 μm for PS concentrations ranging from 10–50 wt.% for rapidly cooled injection molded specimen at 180 ${}^{\circ }$C.

Diaz et al. [79] report an average diameter of 1.42 μm for the surfaces of cryo fractured PP–PS 80:20 blend surfaces prepared in a batch mixer at 200 ${}^{\circ }$C. Rajkiran et al. [80] produced injection molded PP–PS specimens in a twin screw extruder at 220 ${}^{\circ }$C melt and 40 ${}^{\circ }$C mold temperature using three different molecular weight PP types. In this case, the analyzed fracture surfaces display particle diameters of 1.22 (high MW)–4.21 (low MW) μm for 90:10 blends and 2.32 (high MW)–7.7 (low MW) μm for 70:30 blends.

### 3.5. Mechanical Properties

The combination of tensile and tensile impact testing serves as a good method to evaluate the resistance of a material exposed to dynamic as well as continuously applied load. Regarding the successful compatibilization of polymer blends, an increase in tensile impact strength as well as elongation at break is usually reported to occur as the improved phase adhesion prevents sudden rupture. This is mirrored in the results of this study [29,81,82]. A relationship between tensile properties and extensional rheology is apparent as load is applied in uniaxial direction of the specimen in a solid and molten state, respectively.

**Figure 12:**
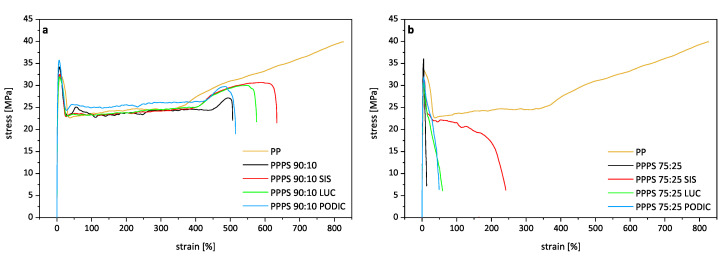
Stress–strain curves for PP–PS 90:10 (**a**) and PP–PS 75:25 (**b**) blends obtained from tensile testing.

Figure 12 illustrates the stress–strain curves obtained from tensile testing for all prepared PP–PS blends and virgin PP. Additionally, the summarized mean values and standard distributions regarding tensile modulus, elongation at break and tensile strength are given in Table 4. It can be observed that the tensile modulus is raised for all blends compared to virgin PP indicating that only a small amount of the highly stiff PS significantly influences the mechanical properties of the compositions. With an increase of PS concentration the modulus is also further increased. While PODIC blends show similar moduli compared to uncompatibilized compositions, the values are slightly decreased for the addition of LUC and SIS. This might either correlate with particle size reduction and improved compatibility as the blend matrix approaches the perfectly homogeneous structure of a virgin material or the presence of more flexible elastomeric interphase between the phases leading to a decrease in stiffness.

Virgin PP exhibits a highly ductile behavior also exhibiting pronounced strain hardening when in a solid state, while the addition of even small amounts of PS are sufficient to result in decreases in elongation at break of almost 50%. With a further increase of PS content, the brittle polymer clearly dominates tensile properties as the uncompatibilized test specimen are rapidly fractured. This might be an indicator for the 75:25 blends being close to beginning phase inversion leading to less stable co-continuous morphologies which are reported by some authors to already occur at PS concentrations around 30–40% [83,84]. Qualitatively compared behavior is also in good accordance with the tensile testing results obtained by Brostow et al. and Gao et al. [29,85], although it must be noted that differences in testing procedure as well as sample preparation can of course lead to significant differences in testing results. Contrary to the strain hardening visible in 90:10 curves, the 75:25 blends rather show pronounced strain softening. The significant strain hardening effects seen in extensional rheology for 90:10 blends can also be correlated to stress–strain curves as higher stress values can be resisted before rupture compared to uncompatibilized blends which. However, with the addition of compatibilizers ductility is improved and in the case of 75:25 blends, a remarkable increase in elongation at break can be seen especially for SIS as the value is raised more than fifteen-fold in comparison to virgin blend. This can also be correlated with good blend homogeneity observed in microscopy investigations. The tripling of the elongation at break value of the PP–PS 75:25 PODIC blend reaching almost the same value as LUC is rather interesting due to the fact that hardly any changes in rheological behavior or morphology could be observed.

Tensile strength, which is regarding to DIN EN ISO 527-1 defined as the first maximum of the stress–strain curve, is in the case of the examined blends identical to yield strength indicating the transition from viscoelastic to non reversible plastic deformation. For 90:10 blends, a certain trend correlation of yield point shifting towards lower ordinate values of compatibilized blends can be seen to shifting the crossover point of G${}^{\prime }$ and G${}^{\prime \prime }$ in dynamic rheology indicating the frequency dependent transition from elastic dominated to viscous flow behavior of the material in molten state. Compared to virgin PP, the tensile strength is generally reduced for all blend compositions and further decreases with the addition of compatibilizing agents resulting in a minimum value for the 75:25 LUC blend.

**Figure 13:**
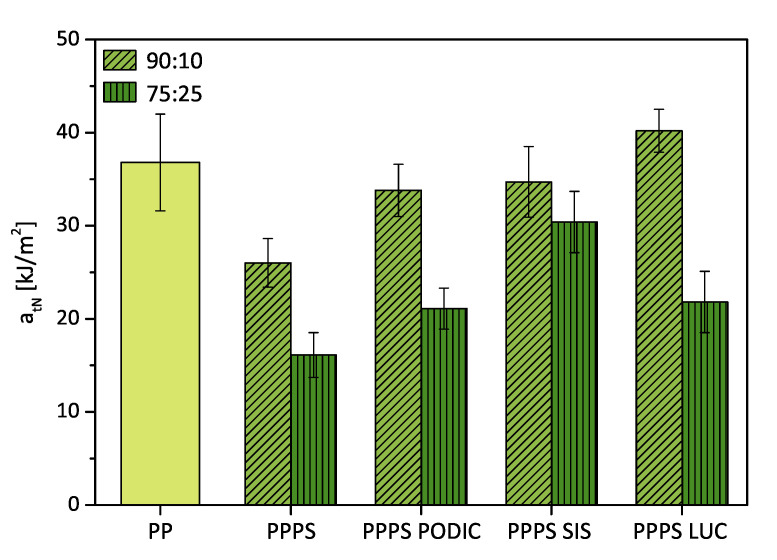
Tensile impact strength of virgin PP and PP–PS blends.

The results from tensile impact testing given in Figure 13 show a good correlation to the trend of ductility increasement seen in tensile testing. While PODIC and SIS are getting close to virgin PP performance LUC even exceeds the value by 15 % for 90:10 blends. Similar to elongation at break the tensile impact strength of 75:25 SIS blend is significantly higher compared to the other compositions doubling the value of the virgin blend. The observed enhancement of impact strength also supports the assumption from morphology studies that improvement of particle monodispersity of particles as observed significantly influences to mechanical behavior.

## 4. Conclusions

Different compatibilizing systems and their impact on the mechanical, rheological and morphological properties of immiscible polypropylene–polystyrene blends, in which PP represents the major phase, were studied. Further, the use of economically viable amounts of added supplements representing only 3% (SIS, LUC) respectively 1% (PODIC) of the overall blend mass as well as industrial applicable processing conditions were considered.

Long chain branches were formed as a result of peroxide induced reactive extrusion with PODIC verified by extensional rheology measurements leading to highly improved melt strength, a slightly finer particle dispersion for the 90:10 blend and a moderate improvement in material toughening. With higher PS concentration, the same tendencies are visible but long chain branching is less pronounced which is also indicated by higher MFR values as a possible result of enhanced chain scission reactions.

Contrary, the addition of non reactive copolymer compatibilizer SIS resulted in the most homogeneous morphology with mean PS particle diameters <1 μm also promoting improved ductility and toughness of blends while melt strength is only raised a little bit. Especially for higher PS concentration, a remarkable increase in elongation at break and tensile impact strength is noticed compared to the other blend compositions.

As a consequence, a combination of both systems seems like a perfect solution to achieve overall best blend performance. Experimental result show that LUC as reactive copolymer is indeed lying somewhere in-between the other two compatibilizing systems but behaving overall less predictable. On the one hand, PP–PS 90:10 LUC exhibits the highest tensile impact strength value even exceeding virgin PP while particle size reduction has not been as effective as in the case of SIS. While on the other hand this behavior is kind of inverted for 75:25 blends in which a finely dispersed morphology similar to SIS is observed but without the corresponding high improvement in impact strength.

Especially at lower PS contents, the reactive extrusion with only a small amount of PODIC seems to be a promising method for the generation of high melt strength blends applicable for manufacturing processes like thermoforming, foaming or fiber spinning. This could also be a promising approach for further investigations relating to the use of post-consumer waste and therefore the generation of innovative recycling material blends.

**Figure 14:**
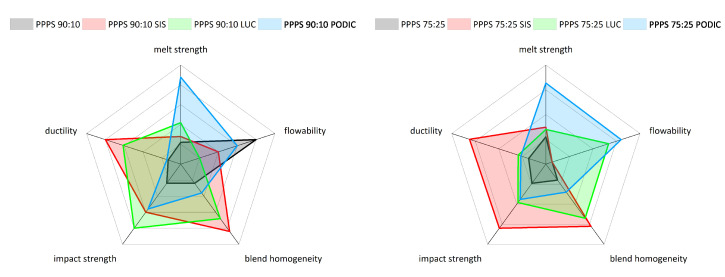
Overall blend performance of the different PP–PS blend compositions related to material testing results.

For a better indication of the overall blend performance, the material properties of all blends related to quantitative experimental results are additionally given in the form of radar charts in Figure 14 for 90:10 as well as 75:25 blend compositions. Ductility and impact strength are related to mechanical blend stability as illustrated in the elongation at break and tensile impact strength values. Rheological behavior is characterized as flowability related to MFR and melt strength reflecting the average of strain hardening coefficients. Blend homogeneity is described using the mean average calculated size of dispersed PS particles.

## Figures and Tables

**Table 1:** Blend composition and specification.

Sample	Composition Specification
PP	virgin PP
PP–PS 90:10	90 wt.% PP–10 wt.% PS
PP–PS 75:25	75 wt.% PP–25 wt.% PS
PP–PS 90:10/75:25 SIS	3 wt.% SIS
PP–PS 90:10/75:25 LUC	3 wt.% LUC
PP–PS 90:10/75:25 PODIC	20 mmol/kg PODIC (1 wt.%)

**Table 2:** Melt flow rate (MFR) values of virgin materials measured at 230 ${}^{\circ }$C/2.16 kg.

Material	MFR (g/10 min)
PP	2.7 ±0.08
PS	2.5 ±0.06
SIS	2.7 ±0.13
LUC	5.7 ±0.11

**Table 3:** Summary of G${}^{\prime }$ = G${}^{\prime \prime }$ crossover point values obtained from dynamic rheology measurements.

Sample	$\omega_{c}$ (rad/s)	$G_{c}$ (kPa)	Interpretation
PP–PS 90:10	50.4	23.5	
PP–PS 90:10 SIS	48.5	19.4	MW ↑ MMD ↑
PP–PS 90:10 LUC	62.6	23.1	MW ↓ MMD ↑
PP–PS 90:10 PODIC	48.1	22.9	MW ↑ MMD ↑
PP–PS 75:25	52.3	21.1	
PP–PS 75:25 SIS	56.5	24.8	MW ↓ MMD ↓
PP–PS 75:25 LUC	52.2	23.8	MW ↑ MMD ↓
PP–PS 75:25 PODIC	53.8	21.7	MW ↓ MMD ↓

**Table 4:** Tensile modulus ($E_{t}$), elongation at break ($\varepsilon_{b}$) and tensile strength ($\sigma_{m}$) of virgin PP and PP–PS blends obtained from tensile testing.

Sample	$E_{t}$ (MPa)	$\varepsilon_{b}$ (%)	$\sigma_{m}$ (MPa)
PP	1616 ± 38	821 ± 3.5	37.0 ± 1.0
PP–PS 90:10	1955 ± 95	520 ± 8.7	34.6 ± 0.6
PP–PS 90:10 SIS	1834 ± 30	620 ± 13.7	32.4 ± 0.3
PP–PS 90:10 LUC	1634 ± 34	592 ± 34.8	31.5 ± 1.0
PP–PS 90:10 PODIC	1948 ± 45	522 ± 10.3	34.5 ± 0.7
PP–PS 75:25	2028 ± 91	14 ± 1.7	35.3 ± 0.5
PP–PS 75:25 SIS	1908 ± 65	234 ± 39	31.7 ± 0.7
PP–PS 75:25 LUC	1810 ± 100	53 ± 9.1	27.3 ± 1.8
PP–PS 75:25 PODIC	2061 ± 115	43 ± 9.3	32.2 ± 1.3
